# 3D Imaging and Measurement of Optic Nerve Sheath Diameter

**DOI:** 10.1167/tvst.15.7.11

**Published:** 2026-07-09

**Authors:** Ronald H. Silverman, Cédric Venuat, Lionel Letoffet, Jeffrey A. Ketterling

**Affiliations:** 1 Department of Ophthalmology, Columbia University Irving Medical Center, New York, NY, USA; 2 Quantel Medical, Cournon d'Auvergne, France; 3 Department of Radiology, Weill Cornell Medicine, New York, NY, USA

**Keywords:** ultrasound, 3D, optic nerve sheath diameter

## Abstract

**Purpose:**

The subarachnoid space between the dura and the pia mater is filled with cerebrospinal fluid, and, with elevated intracranial pressure, optic nerve sheath diameter (ONSD) will consequently increase. ONSD measurement thus serves as a non-invasive means for detecting intracranial hypertension. B-scans, however, only measure ONSD in a plane, and axial symmetry is assumed. In this study, we evaluated a novel ultrasound system for three-dimensional (3D) imaging and measurement of ONSD.

**Methods:**

We imaged both eyes of 18 subjects using 15-MHz B-scans and a prototype 3D system. In this system, a focused single-element transducer was moved in a spiral pattern to sweep out a volume in approximately 5 seconds. We compared transverse B-scan ONSD measurements with values derived from cross-sections of 3D scans.

**Results:**

Mean ONSD was 5.32 ± 0.79 mm by B-scan and 5.57 ± 0.61 mm by 3D. The larger measurement by 3D was borderline statistically significant (*P* = 0.05). The within-eye variability between repeated measurements was comparable. Bland–Altman analysis showed no trend associated with increasing or decreasing ONSD. Eccentricity of 3D ONSD best-fit ellipses to nerve coronal sections averaged 0.51 ± 0.098.

**Conclusions:**

The 3D prototype 5-second acquisition was faster than prior mechanical systems based on single element probes but still somewhat vulnerable to motion artifacts. The ability to assess the ONSD in three dimensions rather than as a transverse cross-section offers an avenue toward improved assessment of nerve anatomy and detection of intracranial hypertension.

**Translational Relevance:**

3D imaging of the optic nerve offers improved assessment of changes associated with elevated intracranial pressure.

## Introduction

The subarachnoid space of the optic nerve is contiguous with the subarachnoid cerebrospinal fluid (CSF)-filled space surrounding the brain.^1^ Consequently, it has long been appreciated that elevated intracranial pressure (ICP) can result in papilledema^2^ and increased optic nerve sheath diameter (ONSD).^1^ Intracranial hypertension may be idiopathic, but it can also result from a number of life-threating conditions, including trauma, tumors, stroke, and encephalitis, among others.^3^

The gold standard for monitoring ICP is an external ventricular drain (EVD), or intraventricular catheter, connected to an external pressure transducer.^4^ ICP can also be assessed by measurement of cerebrospinal fluid pressure via lumbar puncture.^5^ Like EVD, lumbar puncture is an invasive procedure, requiring substantial clinical expertise and an aseptic environment. Although generally safe, it is not risk free, with potential complications including headache, back pain, and, more rarely, cerebral and spinal herniation and infection, among others.^6^

A simple, non-invasive means for detection of elevated ICP is therefore of great importance. Because of the presence of CSF in the subarachnoid space, papilledema and increased ONSD represent potential indicators of elevated ICP.^7^ Although computed tomography (CT) and magnetic resonance imaging (MRI) can provide these measurements, ultrasound is advantageous in terms of simplicity, cost, point-of-care application, imaging speed, and absence of ionizing radiation. Ultrasound measurement of the nerve for assessment of elevated ICP was demonstrated as early as 1987 by Gangemi et al.^8^ Several studies have since shown ultrasound-determined ONSD to be correlated with ICP ex vivo^9^ and clinically as measured by EVD^10^ and lumbar CSF pressure.^11^^,^^12^

A meta-analysis of ultrasound ONSD reported considerable variation in methodology, including head positioning (flat vs. elevated), probe type (linear array, curved array, focused single-element), transducer frequency, mechanical index (MI), and probe placement, and recommendations for standardization to reduce variability were offered.^13^ Variation in marker placement was also notable.^14^ It is generally agreed, however, that measurements should be made 3 mm posterior to the disc where the subarachnoid space is widest.

A recent consensus addressed the element of confusion or disagreement on ultrasound measurement protocols for determination of ONSD.^15^ The recommended protocol calls for transverse imaging parallel to the nerve and measurement 3 mm posterior to the disc. A 7.5-MHz linear array conforming to the U.S. Food and Drug Administration (FDA) acoustic output guidelines for ophthalmic ultrasound (MI ≤ 0.23) was recommended. Also addressed was ambiguity in measurement position, distinguishing between internal and external ONSD (i.e., including or excluding dura thickness). Although these were shown to have equal diagnostic accuracy for detection of intracranial hypertension, they are not equivalent.^16^

It has been reported that ONSD measured in transverse versus sagittal planes varied substantially in a population of intensive-care patients, with sagittal measurement being significantly larger.^17^ This observation suggests a lack of axial symmetry, especially in intensive-care unit patients (many of whom had invasive ICP monitoring in this study).^17^ Asymmetry was also consistent with findings of a recent freehand three-dimensional (3D) ultrasound study of ONSD.^18^

Although measurement in both transverse and sagittal orientations could in part address the issue of nerve sheath asymmetry, the most desirable approach would be obtaining a coronal cross-section of the nerve by 3D imaging. 3D imaging of the eye and orbit has been addressed over the last few decades by a number of approaches: In the 1990s, systems based on the motion of single-element probes were introduced.^19^^–^^22^ More recently, a 3D system with a slit-lamp–mounted mechanical sector B-scan probe was described.^23^ These mechanical B-scan systems are limited in capability, primarily due to their long acquisition time (up to 30 seconds). Faster array-based ultrasound technologies have thus been explored. (The reader should note the “cultural” difference between ophthalmology departments, which almost exclusively use dedicated ophthalmic ultrasound systems with mechanically scanned single-element sector B-scan probes, and virtually all other clinical specialties, which use linear arrays.) Although linear array probe form factor is at times problematic, especially for deep-set eyes, linear arrays are advantageous from the standpoint of imaging speed and depth of field. Nonetheless, they require motion in the elevation axis (normal to the B-scan plane) to obtain 3D data. To bypass mechanical motion systems, freehand 3D imaging of the optic nerve with positional sensors attached to the probe has recently been described.^18^^,^^24^ In this report, we describe a novel, integrated mechanically scanned 3D single-element probe for imaging and measurement of the optic nerve.

## Methods

### Human Subjects

This research followed the tenets of the Declaration of Helsinki and was approved by the Columbia University institutional review board. Informed consent was obtained after explanation of the nature and possible consequences of the study. Eighteen volunteer subjects without a history of ocular disease were recruited (seven male and 11 female). Subject age ranged from 22 to 80 years, averaging 47.7 years.

### 3D Probe and Imaging System

The prototype probe and the imaging system were developed by Quantel Medical (Cournon d'Auvergne, France) in collaboration with Cornell Medicine and the Columbia University Irving Medical Center with National Institutes of Health support. The probe consisted of a 15-MHz, single-element, fixed-focus transducer mounted to a pivot arm within a liquid-filled, sealed housing. The probe was connected to a small electronics control box, and a laptop computer was used to operate the transducer and display data. Transducer acoustic output conformed to FDA guidelines for ophthalmic diagnostic ultrasound (MI ≤ 0.23). Transducer characteristics (from the manufacturer's technical specifications) of the 3D probe were the same as those of the ABSolu 15 MHz B-scan probe (Lumibird Medical, Cournon d'Auvergne, France): 6-mm aperture, 24-mm geometric focus, 12-mm depth of field, 0.115-mm axial resolution, and 0.4-mm lateral beamwidth at the focus. Using a gelatin phantom with a 1% w/v suspension of cross-linked dextran (Sephadex G-150; Cytiva, Marlborough, MA), we measured the ratio of contrast to noise $\left(CNR=\frac{Tissue\;mean-Background\;mean}{Background\;standard\;deviation}\right)$ to be 19 dB in the ABSolu and 13dB in the 3D prototype (which produced some background noise).

**Figure 1.**
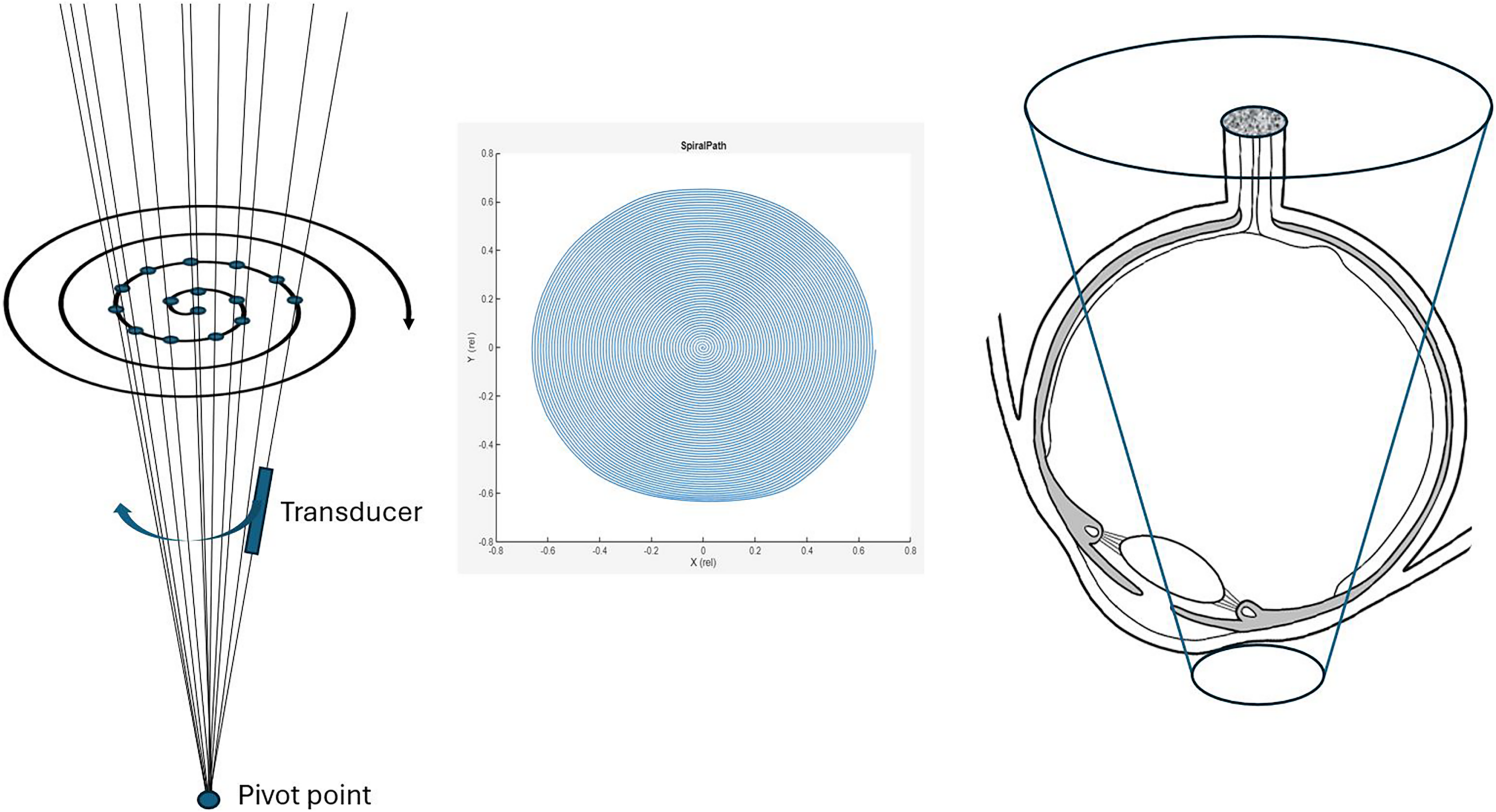
*(* *Left* *)* Schematic illustrating spiral motion of transducer. (*Center*) Transducer position recorded while scanning. (*Right*) The transducer can sweep out a cone-shaped volume in 5 seconds.

As illustrated in Figure 1, during 3D scanning, the transducer moved within the probe housing in an outward-going spiral trajectory, and the orientation of the radial acoustic vectors and echo data were recorded as the transducer moved along its spiral path. This unique scan geometry resulted in a cone-shaped volume with a 40.6° total angular sweep. Each volume, comprised of over 80,000 transmit/receive ultrasound vectors defined relative to a common origin in a spherical coordinate system, was acquired in approximately 5 seconds.

### Scanning Procedure

Scanning and post-processing were performed by one individual (RHS) experienced in ultrasound imaging of the eye. With the subject in a sitting position, both eyes were first scanned with a 15-MHz B-scan probe using a Quantel ABSolu ophthalmic ultrasound system and then with the 3D prototype. In both cases, scans were performed through closed eyelids with the probe acoustically coupled to the eye with GenTeal (Alcon, Fort Worth, TX) ophthalmic lubricant. On the ABSolu, real-time axial B-scans were displayed, and the optic nerve was centered in a transverse orientation. Three cineloops encompassing the optic nerve were acquired on each eye.

Immediately following acquisition of B-scan data, 3D imaging was performed. Real-time B-mode images in a sagittal plane were displayed on the 3D system until the nerve was well centered. 3D scanning was then initiated with a foot-pedal. Three 3D acquisitions were acquired on each eye, after which the scan data were stored, and transverse, sagittal, coronal, and 3D images were displayed, each of which could be manipulated by the user to view planes at different positions through the volume. The volumetric image itself could be rotated, sectioned, and brightness/opacity adjusted.

### Post-Processing and Analysis

**Figure 2.**
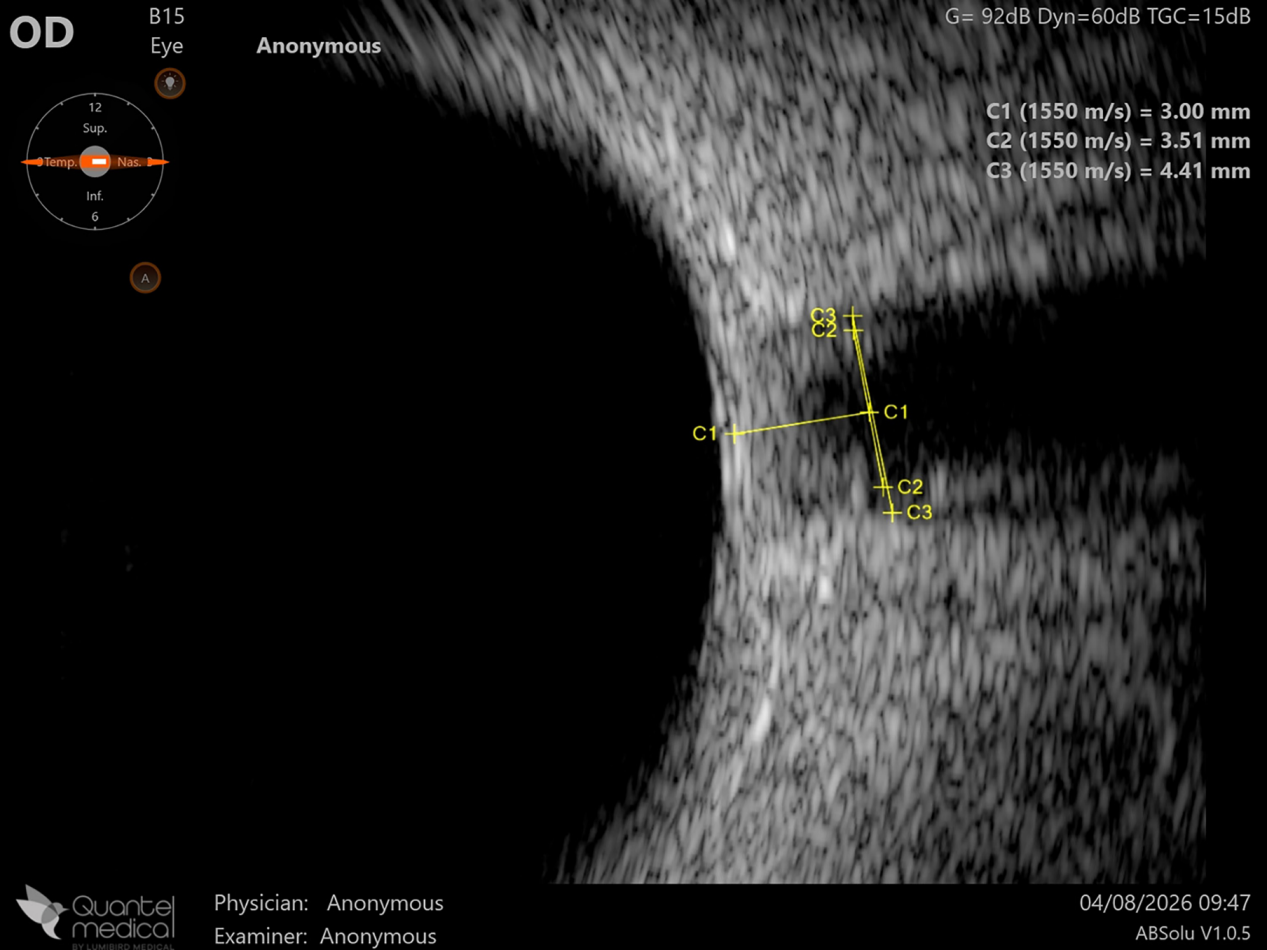
A 15-MHz B-scan of the right eye of normal subject. The scan is in a transverse plane, with measurements of ONSD internal and external dimensions of 3.51 and 4.41 mm, respectively.

ONSD measurements were obtained from de-identified images, with two-dimensional (2D) and 3D measurements performed independently on different days. B-scan cineloop data were reviewed, and the best image of the optic nerve was selected from each sequence. Using ABSolu software, the external optic nerve sheath diameter was then measured 3 mm posterior to the disc (Fig. 2).

**Figure 3.**
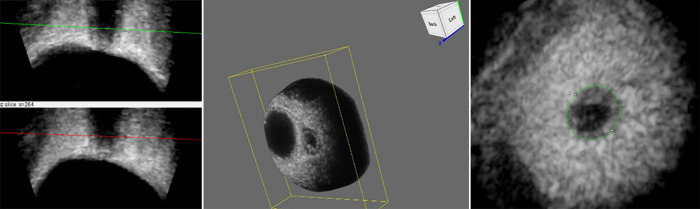
Stages in 3D post-processing. (*Left*) B-mode transverse (*top*) and sagittal (*bottom*) sections passing through the nerve head. Lines drawn through the images depict the manually selected plane normal to the nerve axis. (*Center*) Volumetric image cutting through the orbit to display a coronal cross-section. (*Right*) Section though optic nerve 3 mm posterior to the disc. The best-fit ellipse to external optic nerve sheath diameter is depicted in *green*.

The data from each 3D scan volume were processed with custom software (MATLAB R2025b; MathWorks, Natick, MA) to generate an interpolated stack of 532 coronal 512 × 512 pixel jpeg compressed images with 0.05-mm grid spacing. These were read into Fiji^25^ to produce a 3D volumetric image. To analyze the optic nerve, we identified a plane through the 3D data that was normal to the axis of the optic nerve as follows: A coronal section through the nerve was displayed, and the grayscale image of the nerve and its surrounding was optimized by histogram normalization and applied to the entire 3D stack. Next, a 3D median filter (2 × 2 × 4 pixels in the *x*, *y*, *z* axes) was applied. The data were then read into the Volume Viewer plugin for ImageJ (National Institutes of Health, Bethesda, MD). Sagittal and transverse planes centered on the optic nerve were then displayed, and a plane normal to the nerve in both planes selected and a cross-section of this plane 3 mm posterior to the nerve head were displayed. A series of points around the nerve sheath corresponding to external ONSD were then manually selected, the best-fit ellipse was determined, major (*D_maj_*) and minor (*D_min_*) diameters were recorded, and the mean diameter was computed as *D_mean_* = (*D_maj_* + *D_min_*)/2. The above process is illustrated in Figure 3.

### Statistical Analysis

The transverse ONSD for B-scans was compared to *D_mean_* values obtained by 3D and differences assessed by paired *t*-test and Bland–Altman analysis. Repeated-measures ANOVA was used to determine within-eye variation of ONSD measurements. Also determined was the eccentricity, $e=\;\sqrt{1-\;Dmin^{2}/Dmaj^{2}}$.

## Results

**Figure 4.**
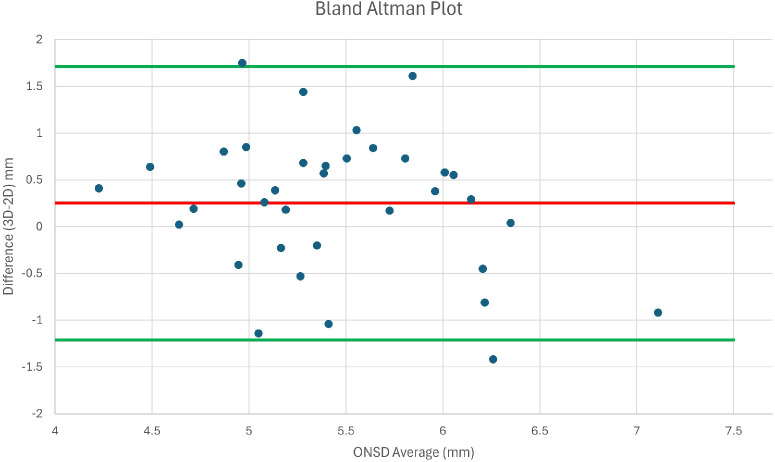
Bland–Altman plot depicting difference between transverse ONSD measurements from B-mode data versus best-fit ellipse ONSD (average of minor and major diameters) from 3D analysis. Mean is depicted in *red*, and the 95% limits of agreement are depicted in *green*.

In B-mode presentations through the 3D data, the axial deviation between the nerve from the acoustic beam axis, which was compensated for in post-processing, ranged from 0 to 10° sagittally and 0° to 7° transversely. Overall, the mean deviation angle was 3.5° ± 2.6°. Mean ONSD was 5.32 ± 0.79 mm by B-scan, ranging from 4.02 to 7.57 mm, and 5.57 ± 0.61 mm by 3D, ranging from 4.43 to 6.65 mm. The mean difference between the B-scan and 3D measurements was 0.25 ± 0.746 mm. This difference was borderline significant (*P* = 0.050). The within-eye SD by B-scan averaged 0.482 mm versus 0.444 mm by 3D. This difference was not statistically significant. Bland–Altman analysis comparing B-mode with 3D (Fig. 4) showed no trend associated with increasing or decreasing ONSD. Eccentricity of 3D ONSD best-fit ellipses ranged from 0.32 to 0.68, averaging 0.51 ± 0.098. By one-sample *t*-test, this was found to be significantly different from 0 (eccentricity of a perfect circle). Its coefficient of variation (CV) for repeat within-eye measurements was 9.2%.

## Discussion

Based on a mechanically scanned single-element probe, the prototype 3D system was very familiar for users of ophthalmic ultrasound systems. Given that it was compared to a B-scan system of the same frequency and focal length, the primary difference was a comparison of ONSD determined from a 2D image sequence (cineloop) versus a 3D volume. By measuring ONSD in three scans with each method, an estimate of method uncertainty could be reached, which was found to be comparable. We found, however, that the 3D estimate of mean ONSD was slightly larger on average (*P* = 0.05) than the 2D value. This may be attributable to eye motion during some scans which would artifactually elongate the nerve cross-section in the motion axis, and/or to true axial asymmetry. Eccentricity of the best-fit ellipse averaged 0.51 (a slightly oval ellipse) and ranged from 0.32 to 0.68. If we make the simplifying (but questionable) assumption that normal ONSDs are axially symmetric, this is explainable as a motion artifact occurring in some instances during the 5-second acquisition. The good reproducibility of eccentricity values (9.2% CV) for repeated within-eye measurements, however, suggests that anatomic asymmetry contributed to the difference between B-scan and 3D estimates of ONSD, rather than this having resulted entirely from a motion artifact.

Although the average difference between 2D and 3D measurements was small (0.25 mm), we found that the variation between 2D and 3D measurements (SD = 0.75 mm) was greater than the within-eye variation (SD_2D_ = 0.48 mm, SD_3D_ = 0.44 mm), suggesting difficulty in identification of the sheath boundary. This is likely a consequence of the degradation of lateral resolution at the depth of the ONSD measurement, 3-mm posterior to the disk, as the probes used a 24-mm focal length fixed-focus transducer. Given the offset of the probe within the housing, the thickness of the eyelid, and the average 24-mm axial length of the normal adult eye, the focus might typically fall ∼3 mm anterior to the disc (and considerably more in myopes). At 3 mm posterior to the disc, the ultrasound beam would be ∼6 mm posterior to the focus. Simulation of the acoustic field^26^ predicts a lateral beamwidth of ∼0.53 mm at this position row-column versus 0.4 mm at the geometric focus, which would degrade definition of the tissue layers (nerve, subarachnoid, and dura).

The prototype system used in this study utilized a novel spiral scan pattern that avoids the potential of unwanted vibrations that would occur with a reciprocating sector motion while also speeding 3D acquisition compared to prior mechanically based systems. As the spiral moves outward from the center, the optic nerve (which is centered in the scan) is hence acquired in a fraction of this duration. Nevertheless, speeding acquisition would be a key factor in improving accuracy and differentiating between true ONSD asymmetry and motion artifact.

Array-based systems are advantageous for the purpose of volumetric imaging in several respects.^27^^,^^28^ An annular array can have a form factor similar to the current prototype single-element probe but would provide an increased depth of field and improved lateral resolution over this depth of field, which would be particularly useful for imaging the nerve and orbit, especially in myopes. A spiral 3D probe utilizing a 20-MHz five-element annular array is now under our evaluation.

Linear arrays offer an order-of-magnitude faster frame rate than mechanically scanned B-scan systems. Ultrafast plane-wave methods offer an additional order of magnitude speed-up beyond this.^29^^,^^30^ Linear arrays also provide improved depth of field via beamforming on transmit and dynamic focusing on receive, which are lacking in fixed-focus single-element probes. Linear array systems are standard for 2D measurement of ONSD in neurology, radiology, and emergency departments. For 3D, however, linear arrays still must be mechanically translated (or tilted or rotated) in the elevation axis to acquire a volume. Although mechanical scanning can be bypassed by freehand probe motion using position-tracking sensors and assembly of B-scans into a 3D volume,^18^^,^^31^^,^^32^ this similarly takes time and does not address the problem of motion artifacts.

3D data, however, may be acquired in milliseconds with a fully populated 2D matrix probe. In such a probe, transducer elements ideally should have a pitch (spacing between elements) of one wavelength (λ) or less to avoid side-lobe artifacts. Consequently, a 15-MHz (λ = 0.1 mm), 1-cm^2^ matrix would require an unmanageable 10,000 independently addressable elements. Although matrix arrays have been an area of interest for many years, the complexity of addressing all of the elements has limited their introduction into clinical use.^33^^,^^34^ A practical approach to bypassing this problem is the sparse array.^35^^,^^36^ In these, however, image quality is somewhat degraded because the full aperture is not employed, and emitted energy is reduced because of the smaller number of elements. An alternative strategy is the row-column array (RCA). This was proposed to address the challenges posed by fully populated matrix transducers and limitations of sparse arrays.^37^ RCAs consist of *N* + *N* superimposed, elongated, orthogonal rows and columns of elements. Instead of the *N*^2^ elements required in a fully populated matrix, the RCA requires only 2*N* elements. The reduced element count can be readily controlled with current technology, and RCAs are well suited to ultrafast plane-wave imaging methods. Although the beamwidth emitted by individual array elements is necessarily elongated, with appropriate transmit/receive sequences lateral resolution can approach the diffraction limit.^38^ Numerous papers describing RCA design and usage, including Doppler depiction of vasculature,^39^^,^^40^ have appeared,^41^ but the eye is still unexplored.

## Conclusions

In this report, we demonstrated a novel ultrasound system for acquisition of 3D data of the eye and orbit. Based on a single-element probe that moved in a spiral scan pattern, acquisition was fast compared to most predecessor single-element 3D technologies. The technology allows measurement of ONSD at arbitrary meridians instead of doing a B-scan and assuming axial symmetry. 3D imaging and biometry thus represent a significant advance with respect to current methods. A limitation of the current study is that both scanning and post-processing were performed by one operator, precluding determination of interoperator reproducibility. Other limitations include the relatively small sample size (18 patients, 36 eyes) and inclusion of only normal subjects. Future studies will focus on papilledema (and pseudopapilledema) and improved technologies. A 3D prototype incorporating an annular array is now under evaluation. Ultimately, matrix probes will be necessary to fully eliminate motion artifacts.
